# Food Safety Perception of the Korean Food Delivery App Users, and Antecedents and Consequences of Trust: Moderating Impact of Hygiene

**DOI:** 10.3390/foods15050949

**Published:** 2026-03-07

**Authors:** Myungken Song, Min Gyung Kim, Joonho Moon

**Affiliations:** 1 Department of Tourism Management, Dong-A University, 2639, Busan 49236, Republic of Korea; mkssong83@gmail.com; 2 College of Business Management, Hongik University, Sejong-si 30016, Republic of Korea; mkim@hongik.ac.kr; 3 Department of Tourism Administration, Kangwon National University, Chuncheon 24341, Republic of Korea

**Keywords:** food safety, food healthiness, green packaging, review information, hygiene, trust, continuance usage

## Abstract

Food safety can be regarded as a critical aspect of consumer protection, and there is a clear need for related research within the context of food delivery apps. In addition, food safety is a multidimensional concept, and its definition may vary depending on the specific context in which it is examined. Therefore, this work investigates food safety in the case of food delivery apps from the perspective of consumers in the Korean market. Food safety was conceptualized through four sub-dimensions: food healthiness, eco-friendly packaging, review information, and hygiene. The study examined the effects of these four factors on trust in food delivery apps and the influence of trust on continuance intention. Also, this work inspects the moderating role of hygiene in the relationship between trust and continuance intention. The survey participants were recruited via an online survey conducted through a professional research firm, yielding 300 valid responses. Hypotheses were tested using structural equation modeling and Hayes’ Process Macro Model 1. The results show that trust is positively influenced by eco-friendly packaging, review information, and hygiene. Additionally, trust significantly affects continuance intention, with hygiene demonstrating a significant moderating effect. This research contributes to the literature by clarifying the definition of food safety in food delivery apps and elucidating the relationships among its key sub-dimensions.

## 1. Introduction

According to Spherical Insights [1], the market size of food delivery apps in South Korea reached US $2.7 billion in 2024 and is projected to grow to approximately US $ 7.0 billion by 2035. In addition, OpenPR [2] reported that the South Korean food delivery app market is expected to grow at an annual rate of around 8%, attributing this growth to the country’s advanced e-commerce infrastructure and technological development. In such a large and rapidly growing market, understanding consumer behavior is crucial for both platform operators and food providers, as it enables companies to respond effectively to market demands and maximize revenue. Based on this context, the research focuses on consumers of food delivery apps in South Korea.

This work employs continuance intention as the dependent variable. Continuance intention has been widely used as a dependent variable in technology-related research [3,4,5]. Given that the present study focuses on a specific system—namely, food delivery apps—continuance intention is considered an appropriate outcome variable. Next, this study incorporates trust as a key construct. Prior research argued that trust is a critical factor in the decision-making process associated with the use of a specific system. Building on this perspective, the present study aims to derive meaningful findings by examining trust within the context of food delivery apps [6,7].

This research adopts a food safety framework. Previous works addressed that food safety plays a significant role in consumer decision-making and serves as a motivating factor that enables consumers to consume food with relief [8]. Scholars further addressed that food safety could be conceptualized as a multidimensional construct, as it is difficult to define with a single term and must be applied differently depending on the case [9,10]. Othman [11] and Leib and Pollans [12] contended that food safety should not be confined solely to hygiene issues, but rather understood within a broader framework that encompasses characteristics capable of protecting consumers’ overall safety. Accordingly, this work operationalizes food safety through four sub-dimensions: food healthiness, green packaging, review information, and hygiene. It indicates that the food safety definition needs to be expanded using various frameworks.

Food is directly linked to individual health, and unhealthy food consumption may lead to adverse outcomes such as obesity; therefore, the intake of nutritious food can be regarded as a form of safe consumption [13,14]. Furthermore, food packaging may contain potential hazards, such as endocrine disruptors and microplastics, which raise growing concerns about their possible health implications. These risks imply that packaging materials could adversely affect consumers when food is consumed directly from them. Accordingly, effective management and regulation of packaging-related risks can be regarded as an essential component of ensuring food safety. In this context, the present study incorporates green packaging as a sub-dimension of food safety, as environmentally friendly packaging materials may serve as a viable solution for health- and sustainability-conscious consumers, which is related to the sustainable development of the platform [15,16,17]. Third, review information is incorporated as a component of food safety. Review information provides consumers with experience-based information from other users and reduces uncertainty in food consumption decisions [18]. For this reason, review information is considered an important food safety factor in this study [18,19]. Lastly, the current study includes hygiene as a sub-dimension of food safety, as unhygienic food may pose risks to consumers’ health, making hygiene a fundamental component of food safety [20,21]. Based on these considerations, this study examines how the four sub-dimensions of food safety influence trust in food delivery apps.

This work investigates the moderating effect of hygiene on the relationship between trust and continuance intention. Food delivery apps are characterized by consumers’ inability to observe the food preparation process at partner restaurants, which may generate consumer anxiety [22]. When consumers experience hygiene concerns, they tend to use the platform as a communication channel to resolve related issues [23,24]. Thus, it can be inferred that the extent to which trust influences continuance intention may vary with consumers’ perceptions of the hygiene of the food provided by partner restaurants because such management can be perceived by consumers as a function of food delivery apps.

All things considered, the first objective of this study is to conceptualize food safety within the context of food delivery apps. Second, this work is to scrutinize the structural relationships among food safety sub-dimensions, trust, and continuance intention. Third, the goal of this work is to identify the moderating effect of hygiene on the relationship between trust and continuance intention. By clarifying the concept of safety in food delivery apps and elucidating the relationships among main variables, this study contributes to the theoretical advancement of the literature. Based on the findings, this research is also likely to provide practical insights that may be useful for managerial decision-making.

## 2. Literature Review and Research Hypotheses

### 2.1. Continuance Usage

Continuance intention refers to an individual’s intention to continue using a specific service or system [25,26,27]. Raza et al. [27] employed continuance intention as a dependent variable to examine the behavior of food delivery app users because repeated usage increases the market share of the business. Similarly, Foroughi et al. [4] investigated the determinants of continuance intention in the context of food delivery apps, addressing that continuance usage is a sort of loyalty behavior. Earlier, Foroughi et al. [3] explored the antecedents of continuance intention in mobile banking, while Li and Wang [5] examined attributes influencing continuance intention toward chatbots in e-commerce. Dağhan and Akkoyunlu [28] used continuance intention as the dependent variable to explore the decision-making patterns of online learners, and Abdul-Halim et al. [29] researched the determinants of continuance intention among mobile wallet users. Collectively, these studies demonstrate that continuance intention is widely employed as a dependent variable across various domains to explain user behavior.

### 2.2. Trust

Scholars defined trust as the degree of belief formed based on positive evaluations of a specific product [6,30]. Aureliano-Silva et al. [24] chose trust as a variable to empirically explore the perceptions of food delivery app users because a credible system increases the likelihood of choosing a platform service. Song and Shin [31] used trust to explain consumer behavior in e-commerce, while Koc et al. [6] investigated consumer characteristics related to halal products with trust as a primary variable. Plus, Seridaran et al. [7] selected trust as a mediator to study user behavior in mobile application systems. Collectively, these works demonstrated that trust is widely utilized as a main element in research across different contexts. Hence, this research adopts trust to figure out the food delivery service digital platform users’ behaviors.

### 2.3. Food Safety and Its Sub-Dimensions

Food safety refers to consumers’ perceptions of the extent to which a product assures that food can be consumed without concern [32,33]. Because food consumption is directly related to individual health, consumers place significant importance on it [33,34]. Leib and Pollans [11] argued that the traditional definition of food safety tends to be interpreted narrowly, focusing primarily on hygiene, and therefore emphasized the need to expand this perspective to encompass a broader range of related issues. Othman [12] defined food safety as a relatively broad concept, referring to the consumption of food in a manner that does not cause harm to consumers. Moreover, previous research stated that food safety perceptions can vary by consumption context and need to be understood as a multidimensional construct rather than measured by a single factor [9,10]. For instance, Kasmain et al. [35] found that among food delivery app users, hygiene, food contamination, and improper cooking temperatures were all associated with food safety. Similarly, Pillai et al. [36] claimed that in the context of food delivery apps, consumers are likely to confront potential risks due to insufficient information about food preparation processes and handling of ingredients. For these reasons, it is necessary to investigate multiple aspects of food safety tailored to the context of food delivery apps.

The first area is food healthiness. Individual health is closely related to food consumption, and with rising living standards, consumers increasingly value maintaining their health through dietary choices [37,38]. In this regard, Alsubhi et al. [39] addressed that consumers are willing to pay a premium for healthy food, while Cammarelle et al. [14] emphasized that health-promoting aspects of food are becoming increasingly important to consumers. Nordhagen et al. [40] alleged that food safety extends beyond the prevention of immediate harm and should also encompass the provision of adequate nutrition and the promotion of positive long-term health outcomes. Alae-Carew et al. [13] noted that consumers in the UK place growing importance on healthy food, particularly that is rich in nutrients. Furthermore, previous research revealed that consumers’ perceptions of the nutritional benefits of food play a critical role in shaping their behavior and decision-making processes [41,42]. The second piece of food safety is green packaging. Singh et al. [43] documented that food safety is closely related to food packaging, emphasizing that packaging plays a crucial role in protecting food from substances that may cause contamination. Green packaging refers to consumers’ perceptions of products that use packaging materials designed to minimize environmental pollution [44,45,46]. In the context of food consumption, consumers are increasingly aware of potential risks, such as exposure to endocrine-disrupting chemicals or microplastics, which can be caused by packaging and temperature, making green packaging a possible solution [15,16,17]. Previous literature demonstrated that food consumers tend to form more positive perceptions of products when green packaging is used [16,47,48].

The third domain is the review information. Riaz et al. [49] and Ramesh et al. [50] claimed that the primary function of food delivery apps is to provide information that helps consumers make purchasing decisions, enabling them to reduce uncertainty based on others’ consumption experience. Wen et al. [51] and Lee et al. [18] documented that the apps’ functions also include offering services that provide food tailored to consumers’ preferences. Shah et al. [52] emphasized that review information based on other consumers’ experiences plays a critical role in helping users assess the utility of the system. Allah Pitchay et al. [53] noted that the quality of information is an important factor in explaining users’ attitudes toward food delivery apps. Similarly, Chen McCain et al. [19] and Shroff et al. [54] alluded that review information in food delivery apps, which combines evaluations of both the food itself and the service, provides a means for consumers to reduce perceived risks based on the experiences of others. Information obtained from other consumers can minimize consumers’ perceived financial risk [12,19]. Therefore, in a broader sense, such information contributes to safety by reducing factors that may cause harm to consumers. The last element is hygiene. Hygiene refers to the state in which food is properly managed to prevent contamination, and since poorly maintained hygiene can pose risks to consumer health, it plays a critical role in consumer decision-making [55,56]. Ratasuk [21] disclosed that consumers’ perceptions of hygiene significantly influenced their behavioral intentions in the context of street food. Mullan and Wong [57], drawing on the theory of planned behavior, confirmed that hygiene plays an important role in shaping consumers’ perceptions of food. Frempong et al. [20] reported that street foods with well-administered hygiene received more positive appraisals from consumers, while Wang et al. [56] revealed that positive assessments of hygiene management were associated with higher ratings in the context of food delivery applications.

### 2.4. Hypothesis Development

Hoyos Vallejo and Chinelato [58] stated that food safety is an essential attribute to build a positive perception of consumers because food affects individual health conditions in either positive or negative ways. Ratasuk and Gajesanand [8] demonstrated a positive influence of food safety on trust. Conversely, Chon et al. [59] showed that a suspicious perception of food safety undermined consumer trust among Chinese consumers. Yee et al. [60] disclosed a positive impact of food safety on trust in the domain of the livestock farmers’ goods. Hence, it can be inferred that food safety could play an imperative role in building trust. Thus, the following hypotheses are proposed:

**Hypothesis 1** **(H1):** *Food healthiness positively impacts trust in the area of food delivery app consumers.*

**Hypothesis 2** **(H2):** *Green packaging positively impacts trust in the area of food delivery app consumers.*

**Hypothesis 3** **(H3):** *Review information positively impacts trust in the area of food delivery app consumers.*

**Hypothesis 4** **(H4):** *Hygiene positively impacts trust in the area of food delivery app consumers.*

Sharif and Barua [61] found a positive effect of trust on continuance usage in the domain of food delivery apps because trust is a condition that minimizes uncertainty in the decision-making process. Also, Mai and Nguyen [62] researched the users of the Uber Eats system, and the findings indicated a positive association between trust and continuance usage. Moreover, Seridaran et al. [7] uncovered that trust positively impacted continuance usage of the mobile application system. Hence, it could be inferred that trust is likely to work as an antecedent of continuance usage in the case of a food delivery app. Given the literature review, this research proposes the following:

**Hypothesis 5** **(H5):** *Trust positively impacts continuance usage in the area of food delivery app consumers.*

### 2.5. Moderating Role of Hygiene on the Effect of Trust on Continuance Usage with Agency Theory

Scholars noted that the role of food delivery app platforms was to address consumers’ inconveniences and anxieties and to resolve issues between food sellers and consumers, which constituted one of the platforms’ core functions [23,24]. In addition, Al Bayari et al. [63] and Teo et al. [64] emphasized that hygiene played an important role in shaping consumer behavior in food delivery contexts. Rha et al. [22] documented that the inability of consumers to directly uncover the food preparation process in food delivery apps may serve as a source of concern. Teng et al. [65] empirically demonstrated that hygiene could function as a moderating variable and that consumers who perceived hygiene levels as high exhibited behavioral patterns that differed from those who perceived hygiene levels as low. When applied to the area of food delivery apps, these findings suggested that when consumers experienced hygiene-related concerns, they were likely to rely on food delivery platforms to address such issues. Because consumers could not directly observe food preparation processes, reducing hygiene-related anxiety likely enhanced the perceived importance of food delivery app platforms. Agency theory provides a relevant theoretical foundation for this perspective. Individuals generally tend to be risk-averse in decision-making processes, and because agents typically possess more information than principals, principals place importance on mechanisms that reduce this imbalance [66,67]. When information asymmetry exists, individuals seek institutional or structural arrangements to mitigate it, which in turn helps reduce the likelihood of moral hazard on the part of agents [67,68]. In the context of food delivery apps, partner restaurants possess more detailed information about food quality and hygiene practices than consumers. Consequently, consumers may perceive a need for monitoring mechanisms that can oversee and regulate potential moral hazards by these restaurants, and they may recognize this monitoring function as an important role of the food delivery platform. If hygiene management is inadequate, consumers may interpret this as a form of moral hazard, which could further increase the perceived importance of the platform’s regulatory and supervisory role. Based on this discussion, the following hypothesis was proposed:

**Hypothesis 6** **(H6):** *Hygiene significantly moderates the relationship between trust and continuance usage in the area of food delivery app consumers.*

## 3. Method

### 3.1. Research Model and Description of the Measurement Items

**Figure 1:**
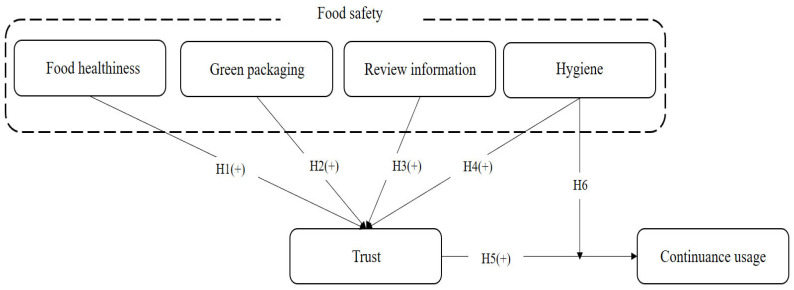
Research model.

Figure 1 is the research model. Food safety has four sub-dimensions: food healthiness, green packaging, review information, and hygiene. Four attributes positively affect trust. Trust also positively influences continuance usage. Hygiene also significantly moderates the relationship between trust and continuance usage.

Table 1 is the description of the measurement items. This work adopted a five-point Likert scale for the measurement of the variables (1 = strongly disagree, 5 = strongly agree). All items were derived from the extant literature, and then they were adjusted to be more suitable for the objective of this work. All items consisted of four attributes. Based on the operational definitions, food healthiness refers to consumers’ perceptions of the extent to which food provided through food delivery apps contributes to their health; green packaging denotes the degree to which packaging used by food delivery apps is perceived as environmentally friendly; review information refers to consumers’ perceptions of other users’ reviews available on food delivery apps; hygiene reflects the extent to which consumers perceive cleanliness to be adequately managed within food delivery apps; trust refers to consumers’ perceptions of the reliability of food delivery apps; and continuance intention denotes the extent to which consumers intend to continue using food delivery apps.

### 3.2. Recruitment of the Survey Participants and Data Analysis

For data collection, this study conducted an online survey. The data were collected through a professional online research firm, Embrain. This work aimed to minimize potential bias in parameter estimates related to gender and age by securing a balanced distribution of observations during the data collection process. Specifically, data were obtained using a convenience sampling approach from participants drawn from a professional research firm’s panel pool [70]. Considering that food delivery apps are widely accessible and utilized across diverse demographic groups, the use of a panel-based sample is deemed appropriate and unlikely to pose a substantial threat to the alignment between the sample characteristics and the objectives of the study. The survey was administered over three days from 19 to 21 January 2026. A total of 300 valid responses were collected, which is considered an adequate sample size to ensure the overall reliability of statistical analyses [70]. The survey targeted consumers in Korea who had prior experience using food delivery apps, and respondents were randomly recruited. This research design reflects the widely accessible nature of food delivery apps and was intended to better capture general market responses. According to Hair et al. [71], when the sample size reaches approximately 300, the assumption of normality tends to be more stable, and such a sample size is considered appropriate for applying structural equation modeling.

Table 2 presents the demographic characteristics of the respondents. The sample consisted of an equal number of males and females (150 each). Respondents were evenly distributed across age groups from their 20s to 50s, with each group accounting for 25% of the sample. Regarding monthly household income, the largest proportion of respondents (39.3%) reported an income between KRW 2.5 million and KRW 5.0 million. In terms of marital status, 156 respondents were single, and 144 were married. Household size was generally well distributed, although four-person households represented the largest share (25.7%). Finally, with respect to weekly usage frequency of food delivery apps, the largest proportion of respondents reported using such apps less than once per week (142 respondents, 47.3%).

This work used Statistical Package in Social Science (SPSS) version 18.0 and Analysis of Moment Structure (AMOS) version 21.0. To analyze the data, confirmatory factor analysis (CFA) was performed to examine the adequacy of the measurement model and to establish convergent validity. Convergent validity was evaluated based on widely accepted criteria: standardized factor loadings of 0.50 or higher, an average variance extracted (AVE) of at least 0.50, and composite reliability (CR) values exceeding 0.70. Model fit was assessed using multiple fit indices, including the root mean square residual (RMR), the normed fit index (NFI), the Tucker–Lewis index (TLI), and the comparative fit index (CFI). Acceptable model fit was determined by an RMR value below 0.80 and NFI, TLI, and CFI values of 0.90 or greater [71,72,73]. Discriminant validity was examined by comparing the square root of the AVE for each construct with the corresponding inter-construct correlation coefficients. Discriminant validity was considered satisfactory when the square root of the AVE for each construct exceeded its correlations with other constructs, in line with established guidelines [71,72,73]. To assess common method bias, this study conducted Harman’s single-factor test. The results showed that the total variance explained was 83.637%, exceeding the threshold of 50% suggested by Hair et al. [71].

Covariance-based structural equation modeling (CB-SEM) was selected to test the proposed hypotheses. Prior research notes that a minimum sample size of approximately 250 is adequate for obtaining reliable parameter estimates in CB-SEM; therefore, the sample size used in this study exceeds this threshold, supporting the robustness of the statistical results. Hypothesis testing was implemented using a 95% confidence interval. In addition, Hayes’ Process macro, which uses 5000 bootstrapping based on ordinary least squares (Model 1), was applied to examine the moderating effect of hygiene without control variables [74]. This is because the study sought to secure a balanced set of observations during data collection in order to minimize potential bias in the estimates related to gender and age for the analysis with no control variable. The simple slope analysis was further employed to illustrate the moderating role of hygiene on the relationship between trust and continuance intention through graphical representation.

## 4. Results

### Convergent Validity and Discriminant Validity

Table 3 is the results of the CFA. The goodness-of-fit indices showed the statistical significance of the CFA (χ^2^ = 478.798 (*p* < 0.01), df = 241, χ^2^/df = 1.987, RMR = 0.030, NFI = 0.935, TLI = 0.961, and CFI = 0.966). All factor loadings and AVE are greater than 0.5, and all CRs are greater than 0.7. All things considered, the convergent validity of the measurement items could be confirmed. Moreover, Table 3 presents the mean values of variables: mean _food healthiness_ = 2.20, mean _green packaging_ = 1.97, mean _review information_ = 2.95, mean _hygiene_ = 2.65, mean _trust_ = 3.08, and mean _continuance usage_ = 3.54.

Table 4 shows the correlation matrix. All diagonals are greater than all off-diagonals, suggesting that the discriminant validity of the measurement items could be ensured. Continuance usage showed the strongest positive correlation with trust (r = 0.630). Trust also positively correlated with green packaging (r = 0.320), review information (r = 0.390), food healthiness (r = 0.327), and hygiene (r = 0.380).

Table 5 is the results of the CB-SEM. Trust is positively affected by green packaging (β = 0.186, *p* < 0.05), review information (β = 0.258, *p* < 0.05), and hygiene (β = 0.204, *p* < 0.05). Also, trust positively impacted continuance usage (β = 0.636, *p* < 0.05). Thus, all hypotheses are supported except for H1.

Table 6 illustrates the results of Hayes’ Process Macro model 1. The model is statistically significant based on the F-value (*p* < 0.05). Trust × Hygiene (β = −0.132, *p* < 0.05) negatively affects continuance usage. Hence, H6 is supported. The R^2^ shows that the model accounts for 41.53% of the variance. The mean values of the low and high hygiene groups are 2.00 and 3.50, respectively.

**Figure 2:**
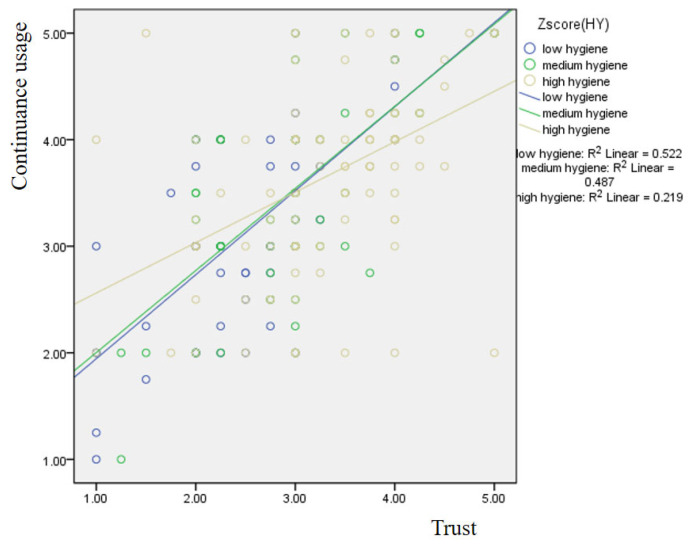
Moderating role of hygiene on the effect of trust on continuance usage.

Figure 2 displays the results of the simple slope analysis. The findings indicate that the slope representing the effect of trust on continuance intention is steeper for the low-hygiene group than for the high-trust group, whereas the slope for the high-hygiene group appears relatively flatter. These results imply that the influence of trust on continuance intention varies depending on the level of trust. In addition, an inspection of the slopes in the graph indicates that there is little difference between the low- and moderate-hygiene perception groups. However, for the group with high hygiene perceptions, the slope representing the relationship between trust and continuance intention appears more gradual.

## 5. Discussion

This research explored the sub-dimensions of food safety from the perspective of Korean consumers using food delivery apps. The sub-dimensions explored were food healthiness, green packaging, review information, and hygiene. In terms of mean scores, eco-friendly packaging recorded the lowest value (1.97), whereas review information had the highest value (2.95). Overall, consumers showed a generally skeptical attitude toward food safety factors, with average scores below 3. The low rating for green packaging likely reflected the tendency of food delivery apps to use inexpensive containers, often made of plastic or Styrofoam. From the perspective of sustainable development, it can be argued that food delivery apps in the Korean market have not achieved particularly positive outcomes in terms of consumer perceptions of green packaging. This may be associated with the financial burden placed on partner restaurants, as the use of environmentally friendly packaging materials can increase costs, potentially leading to higher prices and a loss of price competitiveness in the market. The mean score for food healthiness was relatively low (2.20), which could be attributed to the prevalence of fast-food-type products that are convenient to prepare and deliver while maintaining flavor, as well as the tendency of providers to offer highly flavored foods to survive intense competition. Hygiene received a moderately low score (2.65), possibly due to consumers’ limited visibility into food preparation processes. Review information also scored below 3, which may reflect the presence of biased or incentivized reviews, such as positive ratings influenced by additional fees paid by restaurants.

The research further studied the antecedents and consequences of trust. Food safety sub-dimensions served as antecedents, and continuance intention was treated as the outcome variable. The mean score for trust was 3.08, reflecting a relatively neutral evaluation, whereas continuance intention was higher (3.54), suggesting that habitual use of food delivery apps is likely to contribute to stronger continuance intentions. Food healthiness did not significantly influence trust, which may be explained by the substantial proportion of fast-food-type offerings perceived as less healthy. The current work revealed somewhat different characteristics of food delivery app users compared to Sun and Moon [68]. The discrepancy between the prior study and the present research may be attributed to differences in the study samples, specifically the use of Korean consumers in this study and U.S. consumers in Sun and Moon [69]’s research. In contrast, green packaging positively influenced trust, likely because consumers associated environmentally friendly packaging with health-related factors, such as the absence of endocrine-disrupting chemicals or microplastics, based on the argument of the extant literature [15,16,17]. Review information also positively influenced trust, reflecting consumers’ perception that reviews helped reduce risk in decision-making. The findings are aligned with the outcomes of the prior work because it was found that information delivery is an imperative function of the platform in the case of food delivery apps users’ perceptions [18,51]. Hygiene appeared similarly as a significant variable to account for trust, indicating that well-managed cleanliness positively shaped consumer perceptions of the digital food platform. The findings corroborate previous literature by demonstrating that hygiene significantly influences consumer behavior in the context of food delivery apps, underscoring its importance in food consumption decisions [56,57]. Among the essential factors, green packaging exhibited the weakest effect on trust in food delivery apps. This finding may indicate that green packaging is not yet strongly recognized as a salient attribute of food delivery platforms in the market. Furthermore, compared to attributes such as review information or hygiene—which are more directly associated with potential and immediate threats to consumers’ health—the risks arising from substances released by plastic packaging may be perceived as less tangible or less urgent. As a result, green packaging influenced platform trust appears relatively weaker because this particular attribute is less salient and less directly linked to immediate health concerns.

Trust positively affected continuance intention, indicating that favorable beliefs about a platform encouraged greater usage. Hygiene exhibited a negative moderating effect, indicating that the impact of trust on continuance intention was stronger among consumers who perceived hygiene as low. Hygiene represents a fundamental expectation directly linked to consumer health. When hygiene was perceived as high, users likely judged that risks were already controlled, limiting the additional effect of trust on continuance intention. Conversely, when hygiene was perceived as low, trust functioned as a key attribute in mitigating uncertainty, amplifying its effect. This pattern likely reflected consumer expectations that platforms have to monitor the hygiene practices of partner restaurants, indicating that inadequate hygiene at the restaurant level strengthened the moderating role of hygiene in the trust–continuance intention relationship. Plus, the findings of this work are academically significant in that they provide empirical support for the necessity of mitigating moral hazard arising from information asymmetry between partner restaurants and consumers, particularly by highlighting the governance and monitoring role of the platform in addressing such agency-related problems [67,68].

## 6. Conclusions

### 6.1. Implications

The research has several theoretical implications. First, this work identified four sub-dimensions of food safety—food healthiness, green packaging, review information, and hygiene—from the perspective of Korean consumers in the context of food delivery apps. This study is likely to be significant in that it is based on the premise that previous research on food safety should be examined broadly and that it is necessary to establish a definition appropriate to the specific context [10,11,12]. Second, this study demonstrated that food safety serves as a significant antecedent of trust in this context, while trust, in turn, positively influences continuance intention. The findings of this study can be regarded as valuable in that they provide a thorough examination of trust in food delivery apps. Third, the research revealed that hygiene plays a meaningful moderating role in the relationship between trust and continuance intention, presenting its theoretical relevance. Also, this work is worthwhile in that it applies the framework of agency theory to examine the moderating effect of hygiene.

The findings provide several practical implications. From the perspective of platform operators, although consumers may be somewhat skeptical of review information, it remains an important factor in trust formation. Platforms might need to consider ways to enhance the usefulness of reviews and provide consumers with reliable information. Platforms might also benefit from increasing the proportion of healthy menu options, as consumers are likely to spend more on health-related products; addressing this gap could positively influence trust. Furthermore, platforms might be able to explore strategies to better communicate information regarding eco-friendly packaging and hygiene to consumers. For instance, establishing indices or rating systems to present this information could serve as an effective marketing strategy. Additionally, allocating corporate resources to various trust-building measures—such as ethical management-driven strategies that could foster positive relationships with stakeholders associated with food delivery apps—may further strengthen consumer trust.

For partner restaurants, several practical implications arise. Restaurants that manage hygiene thoroughly might need to actively highlight these efforts through photos or posts to inform consumers of their practices, which builds safety perception. Since consumers tend to rely on the digital platform oversight for hygiene, being recognized as a high-standard restaurant within the platform could be an effective marketing strategy. Restaurants might be able to consider eco-friendly packaging in their operations. For items that are health-related or have environmental implications, emphasizing these aspects in marketing communications can help mitigate potential criticism and appeal to consumer preferences.

### 6.2. Limitations

This research has several limitations. First, this research was implemented in the Korean market, which may limit the generalizability of the findings. Food culture and technological infrastructure vary across countries, so future research might be able to consider diverse cultural contexts. Second, the present study focused exclusively on food safety as an antecedent of consumer trust. Future research should consider incorporating additional antecedents or alternative theoretical frameworks that may offer greater explanatory power in understanding consumer behavior within the context of food delivery apps. Examining variables such as perceived risk, service quality, platform reputation, or technological characteristics could provide a more comprehensive perspective. Finally, this study employed a survey-based methodology. Future research may benefit from adopting more diverse methodological approaches, including big data analytics, experimental designs, and qualitative methods, to gain deeper insights into consumer behavior on digital food delivery platforms. Such approaches could enhance both the robustness and richness of findings in this research domain.

## Figures and Tables

**Table 1:** Measurements items.

Construct	Code	Item	Reference
Food healthiness	FH1	The products offered through food delivery apps contribute to health improvement.	Ruan et al. [48] Sun & Moon [69]
	FH2	The products offered through food delivery apps have high nutritional value.	
	FH3	The products offered through food delivery apps contain high-quality nutrients.	
	FH4	The products offered through food delivery apps are helpful for dieting.	
Green packaging	GP1	Food delivery apps provide environmentally friendly packaging.	Leong et al. [16] Ruan et al. [48] Sun & Moon [68]
	GP2	The packaging used by food delivery apps is not harmful to the environment.	
	GP3	The packaging of food delivery apps is eco-friendly.	
	GP4	The packaging containers used for food delivery do not cause environmental pollution.	
Review information	RI1	User reviews on food delivery apps are trustworthy.	Lee et al. [18]
	RI2	Review information on food delivery apps is reliable.	
	RI3	I believe that the review information on food delivery apps can be trusted.	
	RI4	Review information on food delivery apps is credible for decision-making.	
Hygiene	HY1	I feel that delivered food is generally handled in a hygienic manner.	Mullan & Wong [57] Ratasuk [21]
	HY2	I have rarely felt concerned about hygiene when consuming delivered food.	
	HY3	I feel that the food delivered is prepared in a hygienic environment.	
	HY4	I believe that hygiene standards are well maintained during the food preparation process.	
Trust	TR1	I trust food delivery apps.	Sun & Moon [69]
	TR2	Food delivery apps are reliable.	
	TR3	I think food delivery apps are highly trustworthy.	
	TR4	Overall, food delivery apps are trustworthy.	
Continuance usage	CU1	I will continue to use food delivery apps in the future.	Raza et al. [27]
	CU2	I intend to keep using food delivery apps.	
	CU3	I am willing to continue using food delivery apps.	
	CU4	I have an intention to continue using food delivery apps.	

**Table 2:** Participant characteristics (N = 300).

Characteristics	Frequency	Percentage
Male	150	50.0
Female	150	50.0
20s	75	25.0
30s	75	25.0
40s	75	25.0
50s	75	25.0
Monthly household income		
<$2.5 M KRW	42	14.0
$2.5–5.0 M KRW	118	39.3
$5.0–7.5 M KRW	74	24.7
$7.5–10 M KRW	37	12.3
>10 M KRW	29	9.7
Marital status		
Single	156	52.0
Married	144	48.0
Weekly frequency		
Less than 1 time	142	47.3
1–2 times	135	45.0
3–5 times	20	6.7
More than 5 times	3	1.0
Household size		
1	67	22.3
2	66	22.0
3	69	23.0
4	77	25.7
>5	21	7.0

**Note:** KRW stands for Korean won (Currency rate: $1 is approximately equivalent to 1450 KRW in January 2026), and M stands for million.

**Table 3:** Results of the CFA and descriptive information of attributes.

Construct	Codes	Loadings	Mean (SD)	AVE	CR
Food healthiness	FH1	0.792	2.20 (0.74)	0.660	0.885
	FH2	0.872			
	FH3	0.858			
	FH4	0.721			
Green packaging	GP1	0.808	1.97 (0.83)	0.768	0.929
	GP2	0.885			
	GP3	0.925			
	GP4	0.884			
Review information	RI1	0.898	2.95 (0.84)	0.804	0.942
	RI2	0.927			
	RI3	0.899			
	RI4	0.862			
Hygiene	HY1	0.857	2.65 (0.78)	0.725	0.913
	HY2	0.741			
	HY3	0.903			
	HY4	0.896			
Trust	TR1	0.892	3.08 (0.85)	0.845	0.955
	TR2	0.941			
	TR3	0.921			
	TR4	0.918			
Continuance usage	CU1	0.921	3.54 (0.87)	0.867	0.963
	CU2	0.930			
	CU3	0.921			
	CU4	0.953			

**Note:** AVE, average variance extracted; CR, construct reliability; SD, standard deviation. χ^2^ = 478.798 (*p* < 0.01), df = 241, χ^2^/df = 1.987, RMR = 0.030, NFI = 0.935, TLI = 0.961, and CFI = 0.966.

**Table 4:** Correlation matrix.

Variable	1	2	3	4	5	6
1. Continuance usage	0.931					
2. Trust	0.630 *	0.918				
3. Green packaging	0.191 *	0.320 *	0.876			
4. Review information	0.224 *	0.390 *	0.223 *	0.896		
5. Food healthiness	0.176 *	0.327 *	0.592 *	0.348 *	0.812	
6. Hygiene	0.182 *	0.380 *	0.359 *	0.430 *	0.497 *	0.851

**Note:** * *p* < 0.05, diagonal is the square root of AVE.

**Table 5:** Results of the CB-SEM.

Path	β	Critical Ratio	*p*-Value	Results
Food healthiness → Trust	0.075	0.709	0.478	H1 not supported
Green packaging → Trust	0.186	2.327	0.020	H2 supported
Review information → Trust	0.258	4.205	0.000	H3 supported
Hygiene → Trust	0.204	2.520	0.012	H4 supported
Trust → Continuance usage	0.636	12.318	0.000	H5 supported

**Table 6:** Results of the Hayes’ Process Macro model 1.

Items	β (t-Value)	Results
Intercept	0.619 (1.45)	
Trust	1.017 (7.36) *	
Hygiene	0.339 (2.06) *	
Trust × Hygiene	−0.132 (−2.66) *	H6 supported
F-value	70.09 *	
R^2^	0.4153	
Conditional effects of the focal predictor	Hygiene	
2.00	0.752 (13.07) *	
2.75	0.652 (13.07) *	
3.50	0.553 (8.28) *	

* *p* < 0.05, and the dependent variable is continuance usage.

## Data Availability

The raw data supporting the conclusions of this article will be made available by the authors on request.
